# The non-stop decay mRNA surveillance pathway is required for oxidative stress tolerance

**DOI:** 10.1093/nar/gkx306

**Published:** 2017-05-02

**Authors:** Nur H. Jamar, Paraskevi Kritsiligkou, Chris M. Grant

**Affiliations:** 1 The University of Manchester, Faculty of Biology, Medicine and Health, Manchester M13 9PT, UK; 2 School of Biosciences and Biotechnology, Faculty of Science and Technology, Universiti Kebangsaan Malaysia, 43600 Bangi, Malaysia

## Abstract

Reactive oxygen species (ROS) are toxic by-products of normal aerobic metabolism. ROS can damage mRNAs and the translational apparatus resulting in translational defects and aberrant protein production. Three mRNA quality control systems monitor mRNAs for translational errors: nonsense-mediated decay, non-stop decay (NSD) and no-go decay (NGD) pathways. Here, we show that factors required for the recognition of NSD substrates and components of the SKI complex are required for oxidant tolerance. We found an overlapping requirement for Ski7, which bridges the interaction between the SKI complex and the exosome, and NGD components (Dom34/Hbs1) which have been shown to function in both NSD and NGD. We show that *ski7 dom34* and *ski7 hbs1* mutants are sensitive to hydrogen peroxide stress and accumulate an NSD substrate. We further show that NSD substrates are generated during ROS exposure as a result of aggregation of the Sup35 translation termination factor, which increases stop codon read-through allowing ribosomes to translate into the 3΄-end of mRNAs. Overexpression of Sup35 decreases stop codon read-through and rescues oxidant tolerance consistent with this model. Our data reveal an unanticipated requirement for the NSD pathway during oxidative stress conditions which prevents the production of aberrant proteins from NSD mRNAs.

## INTRODUCTION

Reactive oxygen species (ROS) are ubiquitous molecules formed as a by-product of aerobic metabolism and following exposure to diverse radical-generating compounds. ROS are also generated by neutrophils and macrophages as an important component of immunological defenses against pathogens. For example, hydrogen peroxide (H_2_O_2_) is a ubiquitous molecule which as well as being both freely diffusible and reactive, must be removed from cells to avoid Fenton and Haber–Weiss reactions leading to the formation of highly reactive hydroxyl radicals (1,2). ROS are well known toxicants which damage cellular macromolecules and have been implicated in the cause and progression of many disease processes including cancer, neurodegenerative and cardiovascular diseases (1). To protect against such oxidative damage, cells contain effective defense mechanisms including antioxidant enzymes and free radical scavengers. Despite these protective systems, an oxidative stress occurs when there is an imbalance between radical production and stress protection. Such oxidative stress can result in oxidative damage to most cellular macromolecules and this damage has been implicated in aging and cell death.

It is now well-established that most eukaryotic cells can adapt to ROS by altering global gene expression programs, including those encoding antioxidants and other stress protective systems (3,4). Cells typically respond to stress conditions by invoking complex regulatory mechanisms which act to reprogram translation to respond to the stress. For example, oxidative stress causes a global inhibition of translation which prevents continued global gene expression during potentially error-prone conditions as well as allowing for the turnover of existing mRNAs and proteins while gene expression is reprogrammed to deal with the stress (5). There is also significant mRNA-specific translation of key mRNAs which are required as part of the stress response. The initiation phase is the main target of regulation and represents a key control point for eukaryotic gene expression (6,7). Inhibiting translation initiation is a common response to diverse stress conditions in eukaryotic cells (5). Oxidative stress also causes post-initiation inhibition of translation, increasing the average ribosomal transit time on mRNAs and causing codon-specific pausing (7,8). Oxidative stress conditions affect other stages of translation and for example, have been shown to promote misreading including translational read-through of stop codons and frameshifting (9). Additionally, mRNA turnover is regulated in parallel to transcriptional control as part of the coordinated response to oxidative stress (10,11). Taken together, it is clear that oxidative stress conditions impact protein synthesis at multiple levels and cells must be able to regulate their translational machinery to prevent the production of aberrant proteins.

Oxidative stress has long been known to damage RNA which may functionally affect protein production (12). Not surprisingly therefore, oxidized RNAs are rapidly removed by degradation (13–15). Eukaryotic cells contain quality control systems which monitor mRNAs for errors that might cause the production of aberrant proteins. Cytoplasmic mRNA degradation pathways are initiated by the removal of the poly(A) tail. De-adenylation is then followed by removal of the 5΄-cap and degradation of the mRNA from the 5΄-end by the Xrn1 exonuclease (16). Xrn1 has also been shown to promote general 5΄-3΄ co-translational mRNA decay following the last translating ribosome (8). Alternatively, mRNAs can be degraded from the 3΄-end by the action of the exosome, which is a conserved multiprotein complex that degrades RNAs in the 3΄-to-5΄ direction (17). These degradation pathways are not mutually exclusive and their balance varies depending on the particular mRNA or organism. mRNA decay is linked to translation and there are three translation-associated mRNA surveillance pathways which prevent the production of potentially toxic proteins: nonsense-mediated decay (NMD), no-go decay (NGD) and non-stop decay (NSD). These quality control mechanisms all ultimately target mRNAs for degradation (18). NMD functions to detect premature termination codons (PTCs) and prevents the expression of truncated or erroneous proteins. NGD recognizes transcripts on which ribosomes have stalled during translation, often caused by regions of strong secondary structure (19). NSD is a quality control system for non-stop mRNAs, such as those that lack a termination codon or where ribosomes have by-passed the normal stop codon (20). It recognizes stalled ribosome at the 3΄-end of mRNAs and targets them for rapid degradation.

Given that oxidative stress conditions can directly damage nucleic acids and promote alterations in translational activity, it seems likely that mRNA surveillance pathways might be important during oxidative stress conditions. Consistent with this idea, it has been shown that yeast mutants deficient in the exoribonuclease activity of the exosome are sensitive to oxidative stress (21). Another study has also shown that 8-oxoguanosine (8-oxoG) accumulates in mRNAs isolated from mutants deficient in NGD and the formation of 8-oxoG on mRNAs can stall the translational machinery (15). 8-oxoG is a major nucleotide oxidation product and these data reinforce the idea that mRNA surveillance mechanisms are important to protect against the consequences of decoding damaged RNA. Taken together, these studies suggest that mRNA surveillance pathways might act to prevent oxidative damage to mRNA, or errors in translation, from altering the proteomic output, but this has not been systematically examined. In this current study, we have compared yeast mutants which are defective in various components of mRNA surveillance pathways for their sensitivity to oxidative stress and found that mutants deficient in NSD are particularly oxidant sensitive. To begin to address the mechanisms by which NSD substrates can be generated during oxidative stress conditions, we show that H_2_O_2_ exposure causes the aggregation of the Sup35 eukaryotic release factor 3 (eRF3) termination factor and suggest that the loss of available Sup35 causes an increase in termination codon read-through and ribosomes reaching the 3΄-end of mRNAs to generate NSD substrates. In agreement with this idea we show that increasing the cellular concentrations of Sup35 reduces stop codon read-through and rescues the oxidant sensitivity of a NSD mutant.

## MATERIALS AND METHODS

### Yeast growth conditions

Strains were grown at 30°C (180 rpm) in YEPD media (2% w/v glucose, 2% w/v peptone and 1% w/v yeast extract) or minimal SCD medium (2% w/v glucose, 0.17% w/v yeast nitrogen base supplemented with Synthetic Complete (SC) Kaiser amino acid mixes (Formedium, England). SRaf and SGal media contained raffinose or galactose in place of glucose, respectively. Media were solidified by the addition of 2% (w/v) agar.

### Yeast strains and plasmids

Mutants were constructed in the wild-type yeast strain 74D-694 (*MATa ade1-14 ura3-52 leu2-3,112 trp1-289 his3-200)* including *ski2::HIS3, ski3::HIS3, ski7::HIS3, ski8::HIS3, upf1::HIS3, upf2::HIS3, dom34::HIS3, hbs1::HIS3, ski7::HIS3 dom34::LEU2* and *ski7::HIS3 hbs1::LEU2* using standard yeast methodology. For comparison, *ski2::KanMX, ski3::KanMX, ski7::KanMX* and *ski8::KanMX* mutant were used in the BY4741 background from the Euroscarf yeast deletion collection (22). A strain containing Sup35-GFP was obtained from the yeast Green Fluorescent Protein (GFP) clone collection supplied by Life Technologies. Sup35 was overexpressed using an inducible *GAL1-Sup35* plasmid (23). The levels of UGA termination codon read-through were measured using a dual reporter plasmid which contains tandem *Renilla* and firefly luciferase genes separated by a single UGA stop codon (24).

### Analysis of oxidative stress sensitivity

Stress sensitivity was determined by growing cells to stationary phase in SCD media and spotting diluted cultures (A_600_ = 1.0, 0.1, 0.01, 0.001) onto SCD agar plates containing various concentrations of oxidants. For growth analysis, cultures were initially grown overnight to early exponential phase (A_600_∼0.2) in SCD media before adding H_2_O_2_ to a final concentration of 1 mM. Growth was monitored by measuring A_600_ every hour. For viability analysis, mid-exponential phase (A_60__0_∼0.4) cells grown in SCD media were treated with 2 mM H_2_O_2_ for 1 h. Cultures were serially diluted into YEPD media and plated onto YEPD plates. Viable counts were recorded following 3 days growth and were expressed as a percentage of untreated cultures.

### Polysome and protein analysis

Extracts were prepared in the presence of cycloheximide for polysome analysis as described previously (7). The rate of protein synthesis was measured in exponential phase cells treated with H_2_O_2_. Cells were treated with H_2_O_2_ for 1 h and pulse-labeled for the last 5 min of the treatment with 85μM L-[^35^S] cysteine/methionine as described previously (25).

### Fluorescence microscopy

Cells were washed and immobilized on 10% poly-L-lysine-coated slides. All images were acquired on a Delta Vision (Applied Precision) restoration microscope using a 100×/NA 1.42 Plan Apo objective and fluorescein isothiocyanate from the Sedat filter set (Chroma). Raw images were then deconvolved using the Softworx software and maximum intensity projections of these deconvolved images are shown in the results.

### Protein A-non-stop and stop mRNA assays

The Protein-A NSD (pAV184) and Protein-A stop codon (pAV185) plasmids were a kind gift of Ambro Van Hoof (26). Cells were grown to exponential phase (A_600_ ∼0.3–0.5) in SRaf media before adding 1% galactose, in the presence or absence of H_2_O_2_, to induce expression of the *GAL1* promoter for 6 h. Cells (10 A_600_ units) were broken in 10% trichloroacetic acid with glass beads using a Minibead beater (Biospec Scientific, Bartlesville). Protein A levels were measured using western blot analysis and quantified by C-DiGit® Blot Scanner (LI-COR).

### Protein and western blot analysis

Protein extracts were electrophoresed under reducing conditions on sodium dodecyl sulphate-polyacrylamide gel electrophoresis (SDS-PAGE) minigels and electroblotted onto Polyvinylidene fluoride (PVDF) membrane (Amersham Pharmacia Biotech). Bound antibody was visualized using WesternSure^®^ Chemiluminescent Reagents (LI-COR) and C-DiGit^®^ Blot Scanner (LI-COR). Primary antibodies used were Sup35 (27), Protein A (Sigma) and Pgk1 (ThermoFisher Scientific). Insoluble protein aggregates were isolated as previously described (28,29). Aggregated proteins were visualized by silver staining with the Bio-Rad silver stain plus kit.

### RNA extraction and qPCR analysis

Cells were harvested by centrifugation and resuspended in Trizol (Life Technologies). RNA extraction was performed following the manufacturer's instructions. To assess the levels of actin and Protein A mRNA transcripts, a two-step quantitative polymerase chain reaction (qPCR) method was performed. First, generation of cDNAs was achieved using a ProtoScript^®^ First Strand cDNA Synthesis Kit (NEB) while qPCR analysis was performed using IQ SYBR Green (Biorad).

## RESULTS

### Requirement for mRNA surveillance pathways during oxidative stress conditions

**Figure 1.**
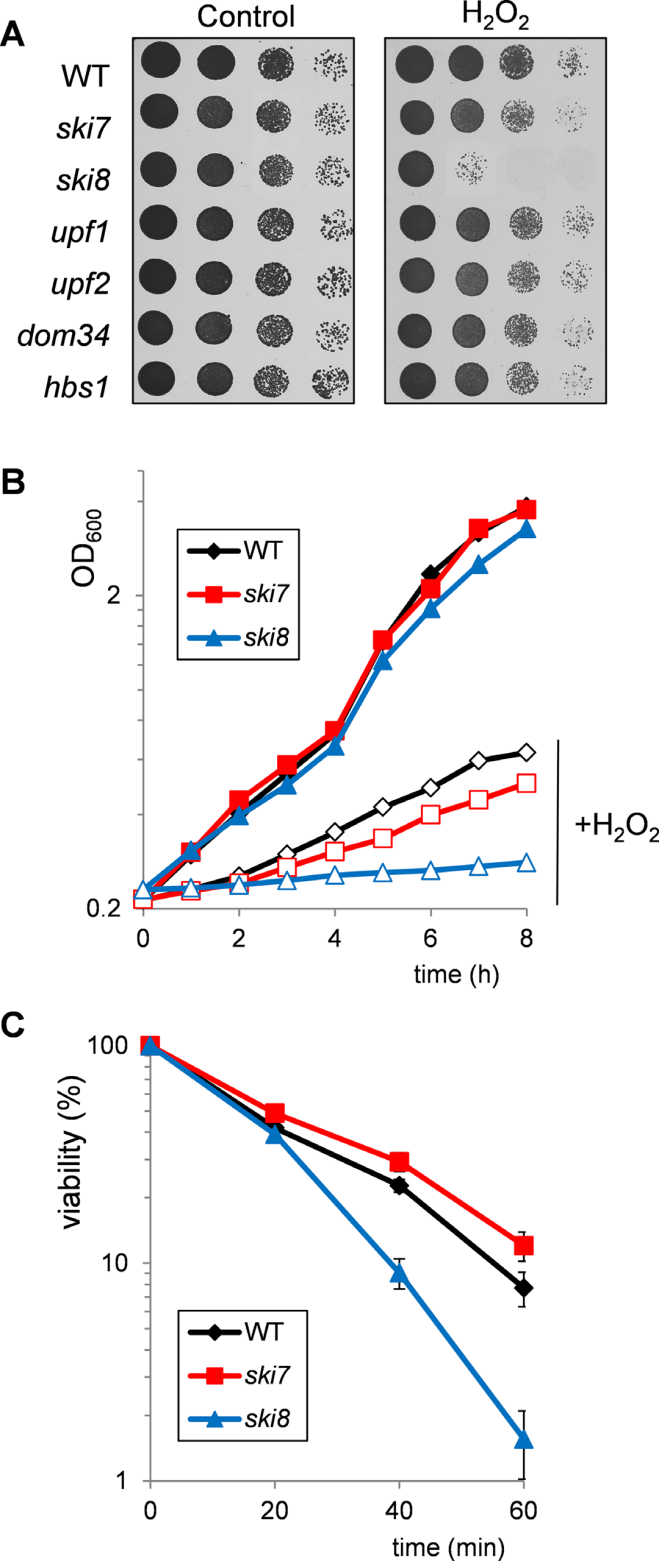
A mutant strain lacking *SKI8* is sensitive to hydrogen peroxide (H_2_O_2_) stress. (**A**) Sensitivity to oxidative stress was determined by spotting strains onto minimal media containing various concentrations of H_2_O_2_. Results are shown for the wild-type, *ski7, ski8, upf1, upf2, dom34* and *hbs1* mutant strains following 3 days growth on 2.5 mM H_2_O_2_. (**B**) Growth curves are shown for the wild-type, *ski7* and *ski8* mutant strains treated with 1 mM H_2_O_2_ for 8 h. Growth was monitored by measuring absorbance at 600 nm. Filled symbols denote growth in the absence of oxidant and open symbols denote growth in the presence of oxidant. (**C**) Viability analysis is shown for the wild-type, *ski7* and *ski8* mutant strains grown to exponential phase in minimal media and treated with 2 mM H_2_O_2_ for 1 h. Cells were diluted and plated in triplicate onto YEPD medium to monitor cell viability. Percent survival is expressed relative to the untreated control cultures.

We compared yeast mutants which are defective in various components of mRNA surveillance pathways for their sensitivity to oxidative stress. Strains were constructed lacking *UPF1, UPF2* (NMD), *DOM34, HBS1* (NGD) and *SKI7, SKI8* (NSD), and their sensitivity to oxidative stress was determined by spotting cultures onto plates containing H_2_O_2_ (Figure 1A). We found that mutants lacking *UPF1, UPF2, DOM34* or *HBS1* were unaffected in H_2_O_2_ sensitivity, suggesting that NMD and NGD are not required for oxidant tolerance (Figure 1A). In contrast, a mutant strain deleted for *SKI8* was hypersensitive to H_2_O_2_ (Figure 1A). However, we found that a mutant lacking *SKI7* was unaffected in sensitivity to H_2_O_2_ compared with the *ski8* mutant.

To confirm the difference in H_2_O_2_ sensitivity between the *ski7* and *ski8* mutants, we examined whether the loss of *SKI7* or *SKI8* alters growth kinetics during oxidative stress conditions. Cultures were grown to the same initial starting density and then exposed to 1 mM H_2_O_2_ before monitoring growth (Figure 1B). Little difference in growth rate was observed between the wild-type, *ski7* and *ski8* mutants in the absence of stress. Exposure to H_2_O_2_ significantly inhibited the growth of all strains, but the *ski8* mutant showed the highest sensitivity compared to the wild-type and *ski7* mutant strains (Figure 1B). This growth sensitivity might arise due to inhibiting cell division or causing cell death. We therefore examined cell viability following a treatment with 2 mM H_2_O_2_ for 1 h. This concentration of H_2_O_2_ is relatively toxic to yeast cells and ∼90% loss of viability was observed in the wild-type and *ski7* mutants within 1 h of treatment (Figure 1C). In comparison, the *ski8* mutant was more sensitive to H_2_O_2_ confirming that H_2_O_2_ causes a greater loss of viability in a *ski8* mutant compared with *ski7* and wild-type strains (Figure 1C).

### Differences in oxidant sensitivity do not arise due to altered control of protein synthesis

**Figure 2.**
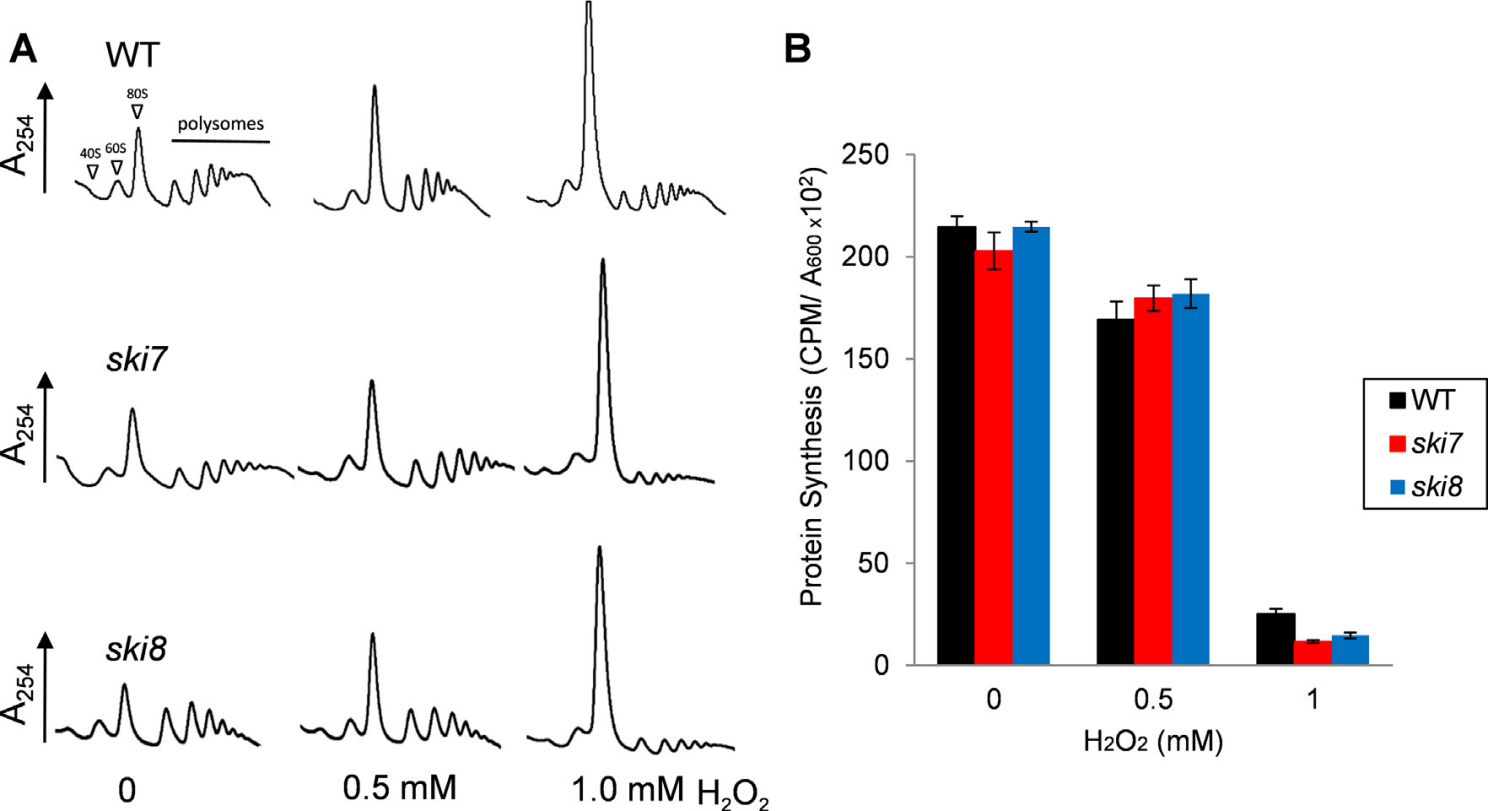
Differences in oxidant sensitivity between *ski7 and ski8* mutants do not arise due to altered control of protein synthesis. (**A**) Polyribosome traces are shown for the wild-type, *ski7* and *ski8* mutant strains treated with the indicated concentrations of H_2_O_2_ for 1 h. The peaks that contain the small ribosomal subunit (40S), the large ribosomal subunit (60S) and both subunits (80S) are indicated by arrows. The polysome peaks generated by 2, 3, 4, 5, etc. A total of 80S ribosomes on a single mRNA are marked with a line. Representative traces are shown from repeat experiments. (**B**) Protein synthesis was measured by pulse labeling cells with [^35^S]-cysteine/methionine for 5 min. Data are shown for untreated cultures (CPM/A_600_) and following treatments with 0.5 or 1 mM H_2_O_2_ for 1 h.

H_2_O_2_ stress is known to cause a global inhibition of translation, predominantly occurring at the initiation phase (5–7). We therefore compared translational activity in wild-type, *ski7* and *ski8* mutant strains using polysome analysis. Extracts prepared from the unstressed strains exhibited peaks corresponding to 40S and 60S ribosomal subunits, monosomes (80S ribosomes) and polysomes (Figure 2A). There was a shift of ribosomes from the polysomal region into the monosome or 80S peak following treatments with 0.5 or 1 mM H_2_O_2_ which is indicative of decreased translation initiation or ribosomal subunit limitation. However, this inhibition was comparable in wild-type and mutant strains. The rate of protein synthesis was measured to further compare translational activity in these strains. Treatment with 0.5 mM H_2_O_2_ modestly inhibited protein synthesis compared with a 1 mM H_2_O_2_ treatment which inhibited protein synthesis by ∼90% (Figure 2B). Again, no significant differences were detected in the level of inhibition in wild-type, *ski7* and *ski8* mutant strains suggesting that alterations in translational activity do not account for the oxidant sensitivity of a *ski8* mutant.

### The Ski complex is required for oxidant tolerance

**Figure 3.**
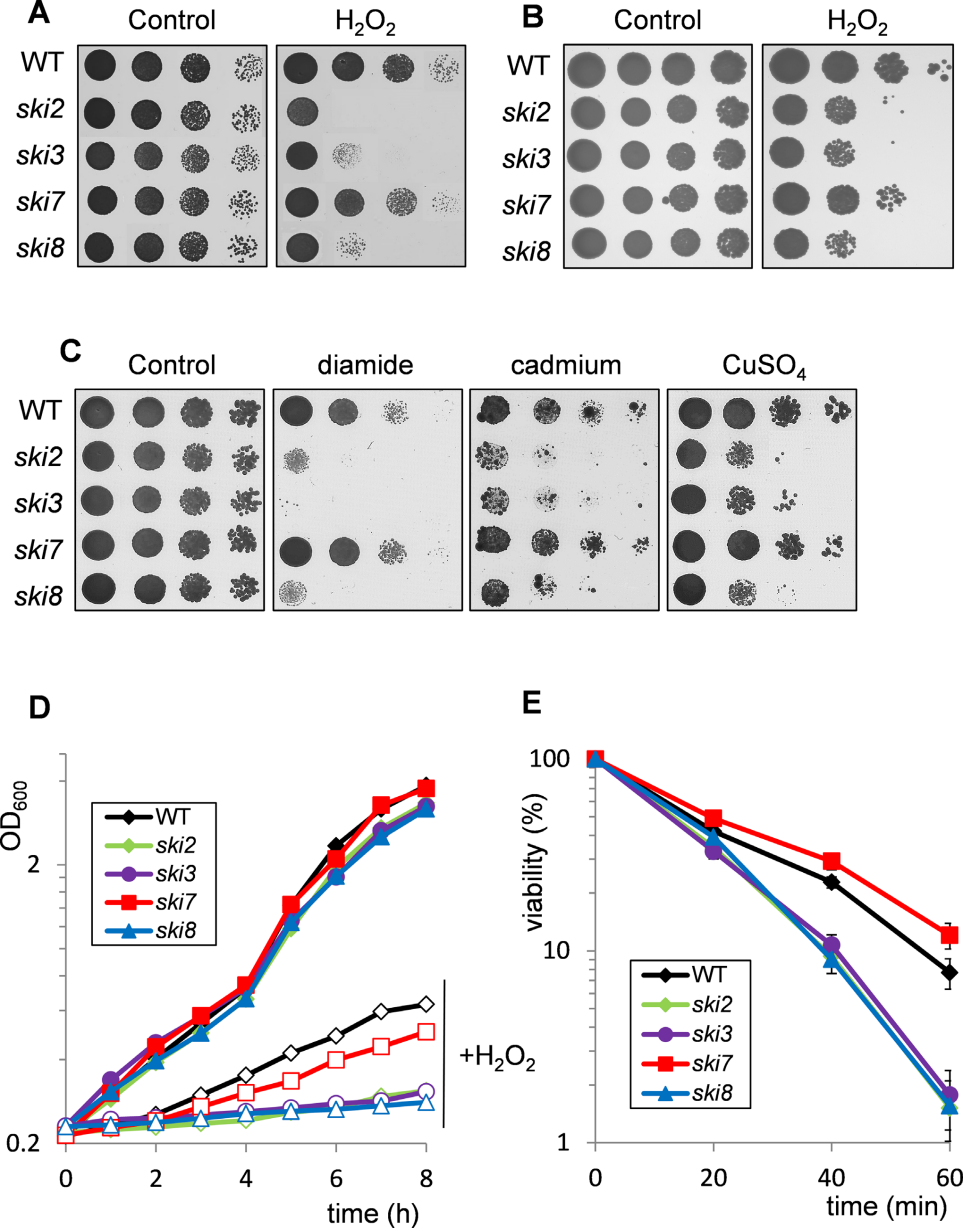
Mutants in the SKI complex are sensitive to H_2_O_2_ stress. (**A**) Sensitivity to oxidative stress was determined by spotting strains onto minimal media containing various concentrations of H_2_O_2_. Results are shown for the wild-type (74D-694), *ski2, ski3, ski7* and *ski8* mutant strains following 3 days growth on 2.5 mM H_2_O_2_. (**B**) Sensitivity to H_2_O_2_ was determined in *ski2, ski3, ski7* and *ski8* mutant strains in the BY4741 yeast strain background, (**C**) Sensitivity to oxidative stress induced by diamide (1 mM), cadmium (20 μM cadmium sulphate) or copper (0.8 mM copper sulphate) was determined in the wild-type (74D-694), *ski2, ski3, ski7* and *ski8* mutant strains. (**D**) Growth curves are shown for same strains treated with 1 mM H_2_O_2_ for 8 h. Growth was monitored by measuring absorbance at 600 nm. Filled symbols denote growth in the absence of oxidant and open symbols growth in the presence of oxidant. (**E**) Viability analysis is shown for the same strains grown to exponential phase in minimal media and treated with 2 mM H_2_O_2_ for 1 h. Cells were diluted and plated in triplicate onto YEPD medium to monitor cell viability. Percent survival is expressed relative to the untreated control cultures.

Ski8 is a component of the Ski complex, which is an evolutionarily conserved complex of three proteins that is functionally and physically associated with the exosome. It comprises the DE*x*H RNA helicase Ski2, tricopeptide repeat protein Ski3 and WD repeat protein Ski8 (20,30). In yeast, Ski7 (an eRF3 family member) bridges the interaction between the SKI complex and the exosome. Ski7 is thought to bind to ribosomes stalled at the 3΄-end of mRNAs where it recruits the exosome to trigger 3΄-to-5΄ degradation of NSD substrates. We therefore tested whether the oxidant sensitivity of a *ski8* mutant is a common feature of SKI complex mutants. We found that mutants deleted for *SKI2* or *SKI3* showed strong sensitivity to H_2_O_2_ similar to a *ski8* mutant as determined using spot test assays (Figure 3A). To check whether this pattern of H_2_O_2_ sensitivity is specific to the 74D-694 yeast strain background, the same *ski* mutants were tested in the BY4741 strain background. Again, the *ski2, ski3* and *ski8* mutants were more sensitive to H_2_O_2_ compared with the wild-type and *ski7* mutant strains (Figure 3B). The sensitivity to H_2_O_2_ appears to be a general sensitivity to oxidative stress conditions since the *ski2, ski3* and *ski8* mutants were also sensitive to diamide, cadmium and copper stress (Figure 3C). Finally, growth rate analysis (Figure 3D) and viability measurements (Figure 3E) confirmed that H_2_O_2_ inhibited cell growth and caused increased loss of cell viability in *ski2, ski3* and *ski8* mutants compared with the wild-type and *ski7* mutants. Taken together, these data indicate that the SKI complex is required for oxidant tolerance, but there does not appear to be a similar requirement for Ski7.

### Analysis of NSD in *ski7* and SKI complex mutants

**Figure 4.**
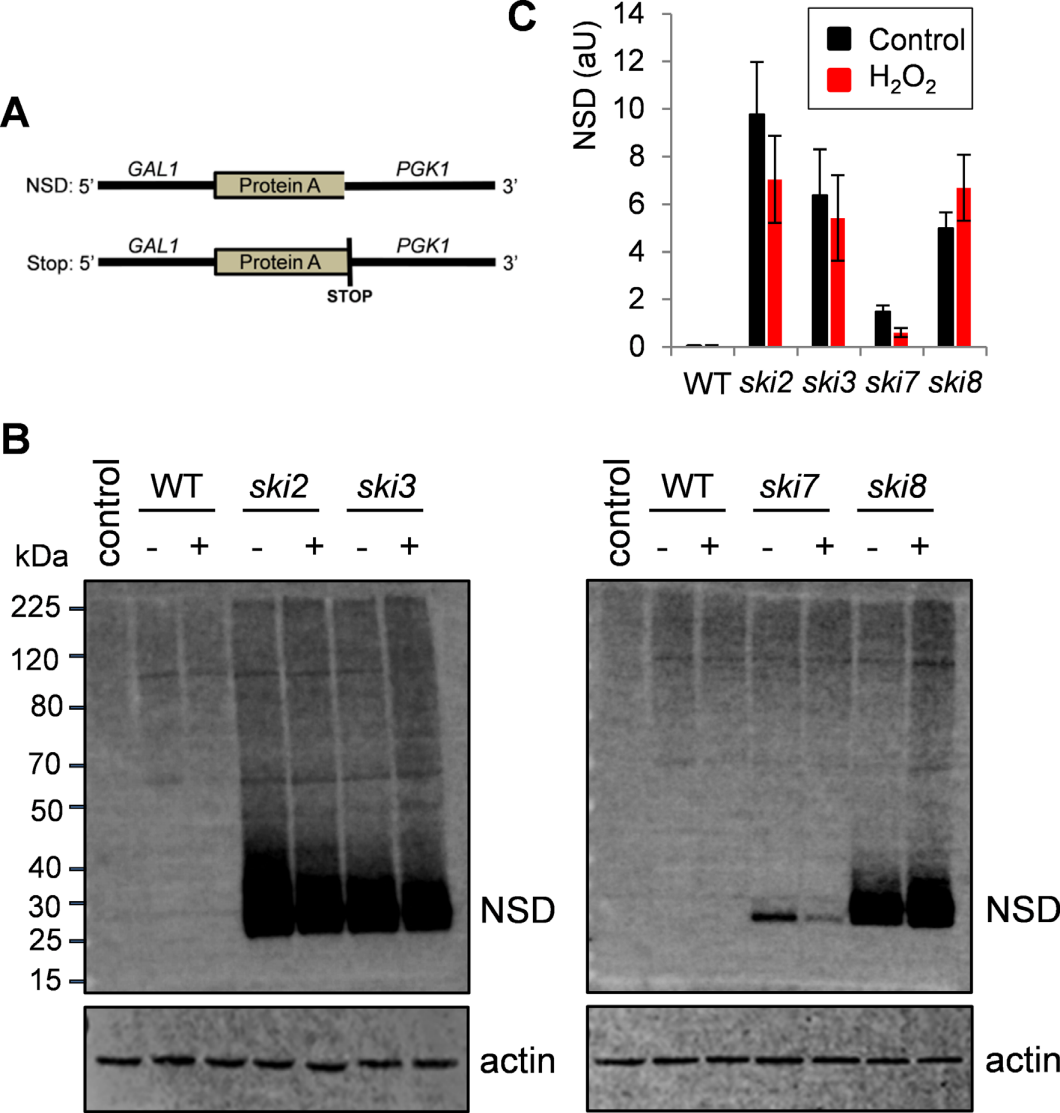
Analysis of non-stop decay (NSD) in *ski7* and SKI complex mutants. (**A**) A Protein A-non-stop reporter construct was used to monitor NSD (NSD construct). This construct contains the *GAL1* promoter, the Protein A coding region lacking its normal stop codon and the *PGK1* 3΄UTR lacking other in frame stop codons (26). A similar construct where Protein A contains its normal stop codon was used for comparison (Stop construct). (**B**) Protein extracts were isolated from wild-type and *ski* mutant strains expressing Protein A-non-stop protein and analyzed by western blotting. Strains were grown in the absence or presence of 0.5 mM H_2_O_2_ for 6 h. Blots were probed with a Protein A antibody (NSD) and an actin antibody as a loading control. Control denotes a wild-type strain containing an empty vector. (**C**) Quantification of Protein A production as shown in panel B from triplicate experiments. Error bars denote standard deviation (SD).

To monitor NSD in *ski* mutants, we used a Protein A-non-stop reporter construct which contains the *GAL1* promoter, the Protein A coding region and the *PGK1* 3΄UTR (26). This reporter lacks the normal Protein A stop codon and downstream stop codons and so the mRNA is a target for NSD (Figure 4A). NSD can be monitored by analyzing Protein A levels which are minimal in a wild-type strain, whereas Protein A should be produced in a NSD mutant due to stabilization of the Protein A-non-stop mRNA (26). We found that Protein A production is very low in a wild-type strain compared with NSD mutants including *ski2, ski3, ski7* and *ski8* (Figure 4B). Interestingly however, far more Protein A was produced in the SKI complex mutants compared with a *ski7* mutant. This analysis was repeated in triplicates and quantified as shown in Figure 4C. We also examined whether oxidative stress caused by H_2_O_2_ exposure affected the production of the NSD Protein A reporter. Oxidative stress did not alter the high levels of Protein A in the *ski2, ski3* and *ski8* mutants, whereas there was ∼50% decrease in Protein A production in the *ski7* mutant. This decrease in the production of an NSD protein correlates with the increased oxidant tolerance of the *ski7* mutant compared with other *ski* mutants. These data suggest that Ski7 is not solely responsible for recognizing NSD substrates in yeast.

### Overlapping requirements for Ski7 and Dom34/Hbs1 during oxidative stress conditions

**Figure 5.**
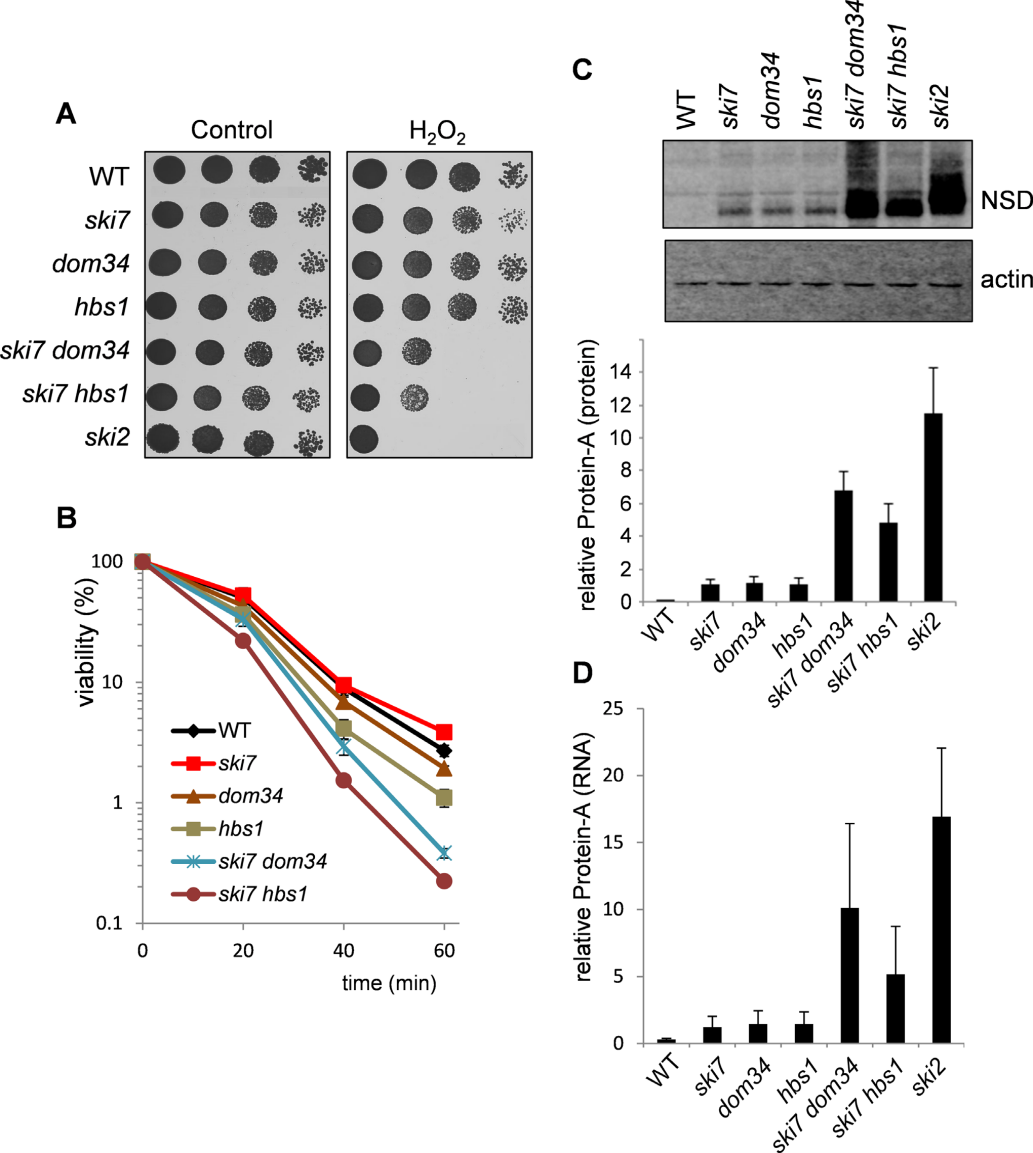
Overlapping requirements for Ski7 and Dom34/Hbs1 during oxidative stress conditions. (**A**) Sensitivity to oxidative stress was determined by spotting strains onto minimal media containing various concentrations of H_2_O_2_. Results are shown for the wild-type, *ski7, dom34, hbs1, ski7 dom34* and *ski7 hbs1* mutant strains following 3 days growth on 2.5 mM H_2_O_2_. (**B**) Viability analysis is shown for the same strains grown to exponential phase in minimal media and treated with 2 mM H_2_O_2_ for 1 h. Cells were diluted and plated in triplicate onto YEPD medium to monitor cell viability. Percent survival is expressed relative to the untreated control cultures. (**C**) Protein was isolated from the wild-type and indicated mutant strains expressing Protein A-non-stop protein and analyzed by western blotting. Blots were probed with a Protein A antibody (NSD) and an actin antibody as a loading control. Quantification is shown for Protein A concentrations relative to actin. Data are the means of three independent biological repeats and error bars denote SD. (**D**) Quantitative RT-PCR of Protein A-non-stop mRNA levels. Quantification is shown for Protein A mRNA concentrations relative to actin mRNA. Data are the means of three independent biological repeats and error bars denote SD.

Ski7 is closely related to Hbs1, which is a conserved family member required for NGD and it is thought that Hbs1 can function in both NSD and NGD (18). We therefore investigated the overlap between NSD (Ski7) and NGD (Dom34/Hbs1) components to examine whether they play a redundant role during oxidative stress conditions. Mutants were constructed lacking *SKI7* and *DOM34* or *SKI7* and *HBS1* and examined for their sensitivity to oxidative stress. Deletion of *DOM34* or *HBS1* increased the oxidant sensitivity of the *ski7* mutant to H_2_O_2_, although the double mutants were not as sensitive as a SKI complex mutant (Figure 5A). We further confirmed the oxidant sensitivity of *ski7 dom34* and *ski7 hbs1* mutants to H_2_O_2_ using viability assays (Figure 5B). This analysis showed that *ski7 dom34* and *ski7 hbs1* mutants were more sensitive to H_2_O_2_ compared with the single mutant strains.

We used the Protein A-non-stop reporter construct to determine whether loss of *DOM34* or *HBS1* affects the recognition of a non-stop mRNA substrate in a *ski7* mutant. The Protein A-non-stop reporter construct was present at similar concentrations in *dom34* and *hbs1* mutants, compared with a *ski7* mutant consistent with the idea that the Hbs1–Dom34 complex can function in NSD (Figure 5C). Furthermore, there was a pronounced increase in Protein A levels in the *ski7 dom34* and *ski7 hbs1* mutants suggesting that Ski7 and the Hbs1–Dom34 complex may play a redundant role in the recognition of NSD substrates. However, the levels of Protein A in the *ski7 dom34* and *ski7 hbs1* mutants were ∼50% of those detected in a *ski2* mutant suggesting that other factors are also required for the Ski complex to recognize NSD substrates (Figure 5C). Quantitative RT-PCR of Protein A-non-stop mRNA levels was used to confirm that the relative concentrations of Protein A-non-stop protein levels correlate with changes in mRNA levels (Figure 5D).

### Why is NSD required for oxidative stress tolerance?

NSD recognizes mRNAs where ribosomes translate into the 3΄-poly(A) tail. This can arise due to processing errors introducing a premature poly-adenylation signal within mRNA coding regions or due to mutations that alter normal stop codons and their recognition (20,31,32). There is no evidence at present to suggest that oxidative stress promotes premature poly-adenylation. It is not thought that mutations in stop codons would routinely generate NSD substrates since frequent in-frame stop codons are found in the 3΄UTRs of eukaryotic mRNAs (31). We therefore considered that conditions which promote nonsense suppression might cause read-through of multiple mRNA stop codons effectively generating NSD substrates. Oxidative stress conditions are known to alter translation efficiency and for example, have been shown to promote misreading including translational read-through of stop codons (9,33,34). The possible mechanisms underlying this increase in read-through during oxidative stress conditions are largely unknown. One possibility is that oxidative stress conditions target the translation termination machinery, since reducing its efficiency would increase the frequency of stop codon read-through. The eRF3 (Sup35) from *Saccharomyces cerevisiae* is well known for its ability to form prion aggregates known as [*PSI^+^*] (35). Formation of Sup35 amyloid aggregates sequesters it away from its normal function in translation termination and elevated read-through of termination codons is therefore a well-known phenotype of the yeast [*PSI^+^*] prion. However, [*PSI^+^*] formation is an extremely rare event occurring at a frequency of ∼5 × 10^−5^ during normal growth, with a 10-fold increase observed in response to oxidative stress conditions (36). Thus, rare prion formation would not cause a measurable increase in stop codon read-through in a population of yeast cells. We therefore asked whether amorphous aggregation of Sup35 occurs in response to oxidative stress conditions, which would deplete Sup35 from the soluble fraction, increasing nonsense suppression and therefore potentially generating NSD substrates.

**Figure 6.**
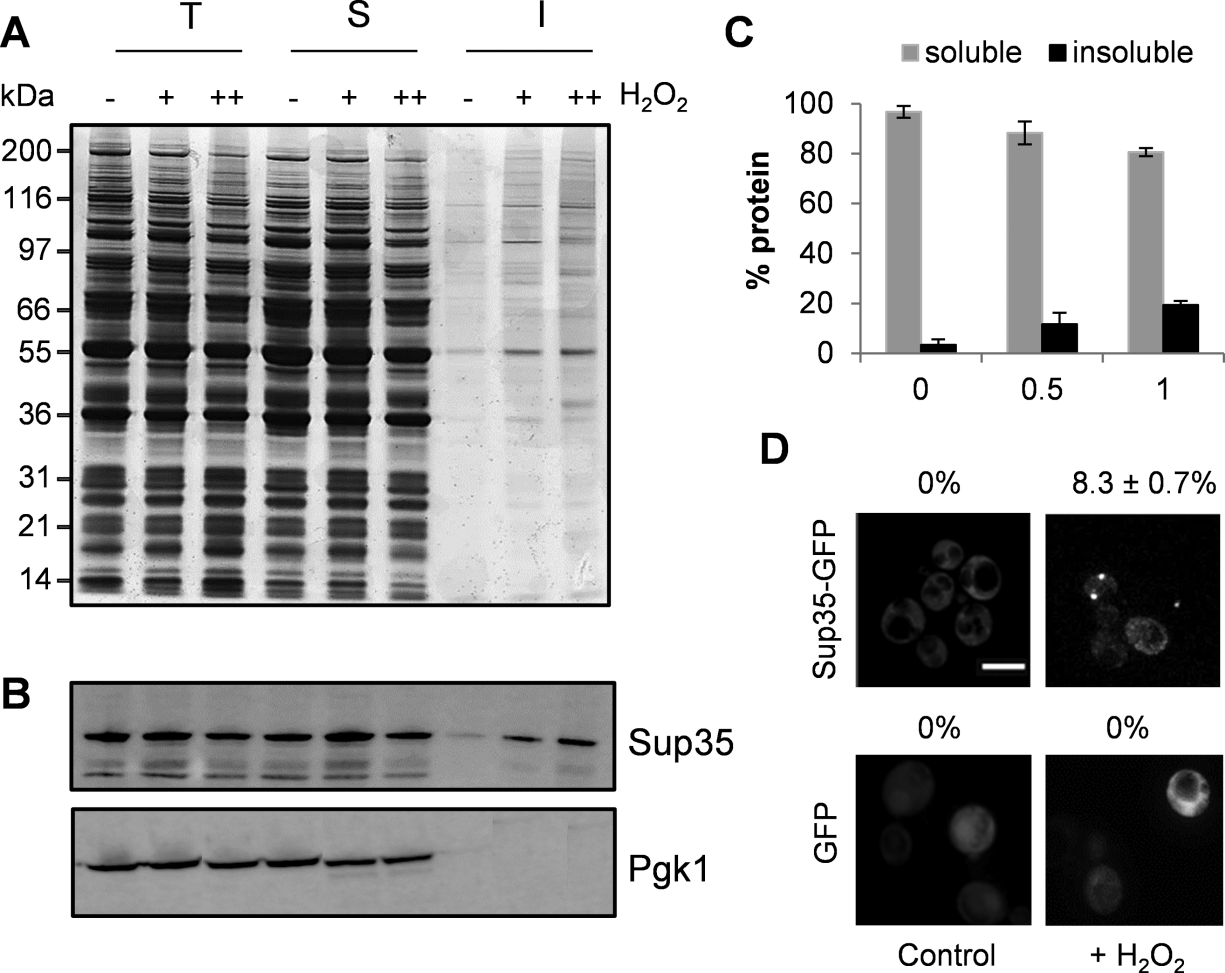
Aggregation of Sup35 in response to H_2_O_2_ stress. (**A**) Protein aggregates were isolated from the wild-type strain grown in the presence of 0.5 mM (+) or 1 mM (++) H_2_O_2_ for 6 h and analyzed by sodium dodecyl sulphate-polyacrylamide gel electrophoresis (SDS-PAGE) and silver staining. T, total cell extracts; S, soluble fraction; I, insoluble aggregate fraction. Four-times as much of the insoluble fraction was loaded relative to the total or soluble fractions to aid visualization. (**B**) The same protein samples as for panel A were analyzed by western blotting using antibodies that recognize Sup35 and Pgk1 as a loading control. (**C**) Quantification of soluble and insoluble Sup35 (percentage of total) as shown in panel B from triplicate experiments. Error bars denote SD. (**D**) Sup35-GFP or GFP was visualized in the wild-type strain exposed to 1 mM H_2_O_2_ for 6 h. Examples of cells containing visible Sup35-GFP puncta are shown. The percentage of cells containing visible puncta was quantified from three independent biological repeat experiments ± SD.

Cellular protein aggregation was analyzed using a biochemical approach which separates insoluble proteins from soluble proteins by differential centrifugation and removes any contaminating membrane proteins using detergent washes (28,37–39). The global levels of protein aggregation were low during control, non-stress conditions but increased in response to treatments with 0.5 or 1 mM H_2_O_2_ (Figure 6A). Western blot analysis of Sup35 revealed that a small fraction of Sup35 was detected in the insoluble fraction during non-stress conditions and this proportion increased in response to H_2_O_2_ stress (Figure 6B). The glycolytic enzyme Pgk1 was used as a negative control and minimal amounts of Pgk1 were detected in the insoluble fraction during normal or stress conditions (Figure 6B). Following the treatment with 1 mM H_2_O_2_ for 6 h, ∼20% of Sup35 was present in the insoluble fraction (Figure 6C). As an alternative means to confirm the aggregation of Sup35 in response to oxidative stress, we examined the distribution of Sup35-GFP using fluorescence microscopy (Figure 6D). Diffuse cytoplasmic Sup35-GFP fluorescence was observed in all cells during non-stress conditions. However, following a 1 mM H_2_O_2_ treatment, ∼8% of cells were found to contain visible fluorescent puncta consistent with Sup35 aggregation. In comparison, no aggregation was observed in a yeast strain expressing GFP alone, confirming that aggregation is due to Sup35 rather than GFP itself.

### Overexpression of Sup35 rescues the sensitivity of a *ski2* mutant to oxidative stress conditions

**Figure 7.**
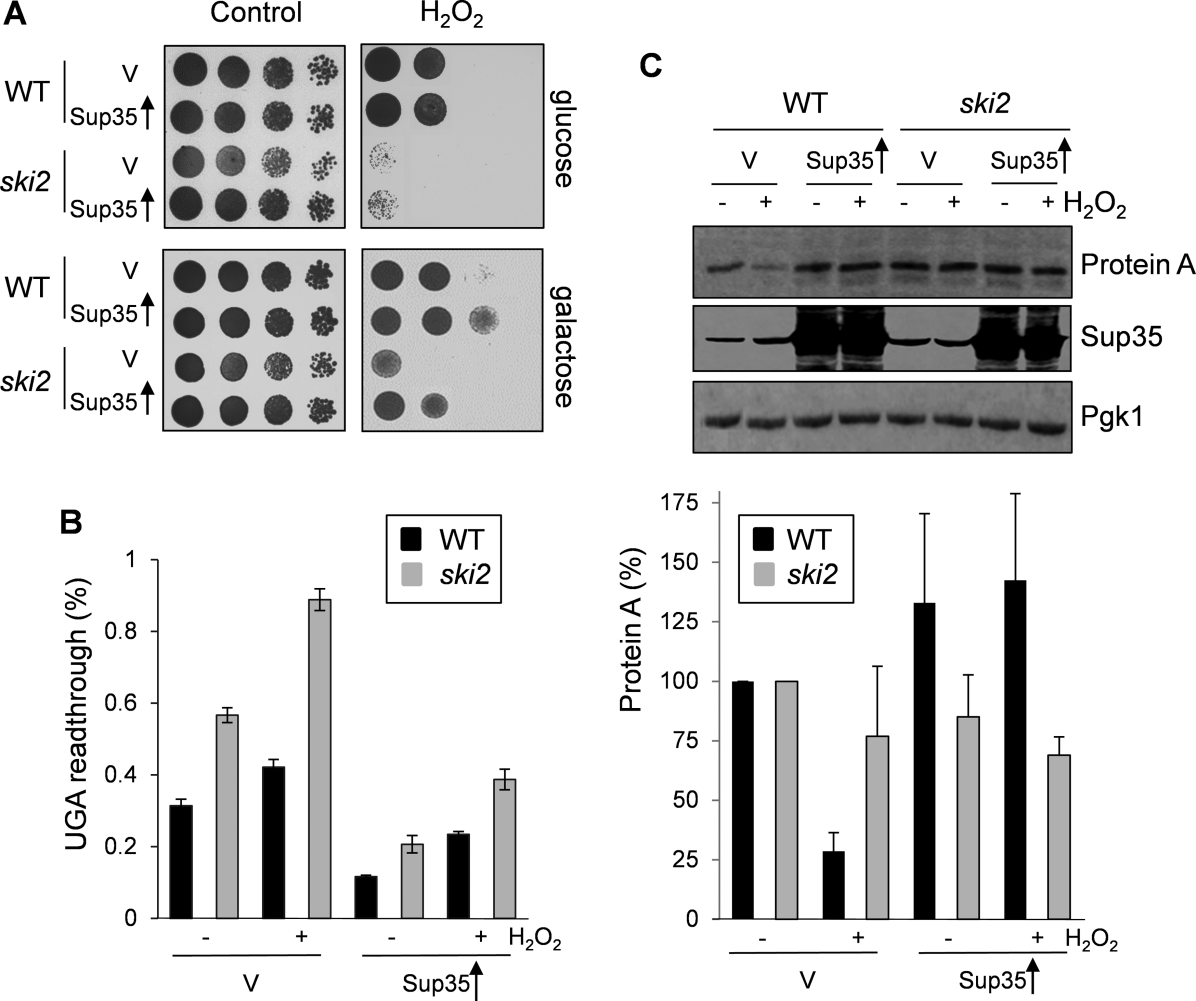
Overexpression of *SUP35* rescues H_2_O_2_ stop codon read-through. (**A**) Sensitivity to oxidative stress was determined by spotting strains onto SRaf or SGal media containing various concentrations of H_2_O_2_. Results are shown for wild-type and *ski2* mutant strains containing an empty vector or overexpressing *SUP35*. (**B**) The levels of termination codon read-through were measured using a dual reporter system in wild-type and *ski2* mutant strains containing an empty vector or overexpressing *SUP35*. Strains were grown to exponential phase in minimal media and treated with 0.5 mM H_2_O_2_ for 6 h. Read-through was quantified using a plasmid that contains tandem *Renilla* and firefly luciferase genes separated by a single UGA stop codon. Values shown are means ± SD from three independent determinations. Read-through is expressed as a percentage of Firefly/renilla. (**C**) Protein was isolated from the wild-type and *ski2* mutant expressing the Protein A-stop construct and containing an empty vector or overexpressing *SUP35*. Western blots were probed with a Protein A antibody and a Pgk1 antibody as a loading control. Blots probed with an anti-Sup35 antibody confirm overexpression of Sup35. Quantification is shown for Protein A concentrations relative to Pgk1. Data are the means of three independent biological repeats and error bars denote SD.

Given that Sup35 aggregates during oxidative stress conditions, we next examined whether increasing the cellular concentration of Sup35 could rescue oxidant sensitivity. For these experiments, Sup35 was expressed under the control of the *GAL1* promoter and the oxidant sensitivity of wild-type and *ski* mutant strains examined using spot test assays. Overexpression of Sup35 was found to improve the H_2_O_2_ tolerance of both wild-type and *ski2* mutant strains, consistent with loss of Sup35 activity accounting for oxidant sensitivity (Figure 7A). Similarly, overexpression of Sup35 rescued the oxidant tolerance of *ski3* and *ski8* mutants (data not shown).

We used two different assays to confirm that oxidative stress increases the read-through of stop codons and that this can be rescued by the overexpression of Sup35. Read-through of termination codons was first quantified using a dual reporter construct which contains tandem *Renilla* and firefly luciferase genes separated by a single UGA stop codon (24). The level of UGA termination codon read-through was elevated in a *ski2* mutant strain compared with a wild-type strain and read-through was increased in response to H_2_O_2_ stress in both strains (Figure 7B). Overexpression of Sup35 lowered UGA termination codon read-through in both the wild-type and *ski2* mutant strains during both normal and oxidative stress conditions (Figure 7B). Western blot analysis confirmed that Sup35 was similarly overexpressed in the wild-type and *ski2* mutant strains (Figure 7C). As an alternative approach, we used the Protein A construct containing an in-frame termination codon, which lacks other in-frame termination codons in the 3΄ UTR (Figure 4A). We reasoned that if read-through of the sole stop codon in this construct was increased in response to H_2_O_2_ stress, then ribosomes would translate through to the 3΄-end of the Protein A mRNA resulting in the generation of an NSD substrate. In agreement with this idea, Protein A production was decreased in the wild-type strain in response to oxidative stress, whereas Protein A production was unaffected in a *ski2* mutant consistent with NSD accounting for the loss of Protein A following oxidant exposure (Figure 7C). Overexpression of Sup35 prevented the oxidant-induced decrease in Protein A production in agreement with the idea that loss of Sup35 activity during oxidative stress conditions results in stop codon read-through and the generation of NSD substrates.

## DISCUSSION

Cells contain conserved mRNA surveillance pathways that target mRNAs for degradation to prevent the production of aberrant proteins. We tested the hypothesis that these mRNA surveillance pathways are required to act as quality control systems during exposure to oxidative stress. Mutants deleted for components of the NGD and NMD pathways were relatively unaffected in oxidant sensitivity indicating that defects in the recognition of ribosomal pauses (NGD) or PTCs (NMD) does not result in yeast sensitivity to oxidative stress conditions. In contrast, mutants deleted for components of the SKI complex or for recognition of NSD substrates were hypersensitive to H_2_O_2_. This suggests that the recognition and degradation of NSD substrates is required for oxidant tolerance. The exosome is the main cellular nuclease which catalyzes 3΄–5΄ mRNA degradation and previous studies have shown that mutants deficient in its exoribonuclease activity are sensitive to oxidative stress conditions (21). These data therefore suggest that the inability to recognize and degrade NSD substrates causes sensitivity to oxidative stress, presumably as a result of the translation and production of aberrant proteins from NSD mRNAs.

The mechanism of NSD is conserved among eukaryotes with the exception of Ski7 which is only found in a small subset of yeasts and not in higher eukaryotes (40,41). Ski7 bridges the interaction between the SKI complex and the exosome (20). Ski7 binds to ribosomes stalled at the 3΄-end of mRNAs where it recruits the exosome to trigger 3΄-to-5΄ degradation of NSD substrates. Ski7 is closely related to Hbs1, which is another conserved Sup35 family member required for NGD and it is thought that Hbs1 can function in both NSD and NGD (18). The Dom34–Hbs1 complex binds to the ribosomal A site and promotes the dissociation of subunits on stalled ribosomes (42,43). This is important for RNA quality control in NGD targeting aberrant mRNAs for degradation by the exosome (19). The Dom34–Hbs1 complex is also thought to play a role in NSD by facilitating the degradation of mRNAs where ribosomes are stalled at the 3΄ end of mRNAs lacking a termination codon (44). This is because the translation of poly(A) tails into poly-lysines following stop codon read-through can cause stalling analogous to NGD (45). The Hbs1–Dom34 complex has also been directly shown to function in NSD in mammalian cells (46). We used a Protein A-non-stop reporter construct (26) to monitor NSD and found that Protein A production was higher in *dom34* and *hbs1* mutants, compared with a wild-type strain, consistent with a role for the Hbs1–Dom34 complex in the turnover of NSD substrates. Furthermore, Protein A production was increased in *ski7 hbs1* and *ski7 dom34* mutants compared with the single parent mutants in agreement with the idea that Ski7 and Hbs1–Dom34 play a redundant role in the recognition of NSD substrates.

Most studies on NSD have used artificial reporter constructs similar to the Protein A-non-stop reporter construct (26) used in our current study since relatively little is known regarding physiological NSD substrates. The most likely source of NSD substrates is considered to arise from 3΄-end processing signals occurring within gene coding regions causing premature 3΄-end cleavage and processing (31). While this would occur during normal growth conditions, it is also possible that oxidative stress conditions might cause cleavage and mRNA truncation events similar to defective 3΄-end processing events. Stop codon read-through has also been considered as a potential source of NSD substrates, but the prevalence of in-frame stop codons in the 3΄UTRs of most mRNAs means that it was considered unlikely as a major source of NSD substrates (31). For example, in-frame stop codons are significantly over represented downstream of normal open reading frame (ORF) stop codons (47,48). We hypothesized that stress conditions, such as oxidative stress, which reduce the fidelity of stop codon recognition, might generate NSD substrates by causing ribosomes to read-through stop codons into the 3΄-end of mRNAs. Although stop codon read-through is a relatively rare event, the production of even small amounts of aberrant proteins due to stabilization of carboxyl-terminal extended proteins may be sufficient to cause toxicity. The addition of C-terminal amino acids to just one key protein could potentially alter its biological function potentially resulting in toxicity. For example, previous studies have identified cases where read-through into 3΄UTRs generates aberrant and aggregated proteins which can cause toxicity (48–50). NSD may therefore be particularly important to protect against protein production under oxidative stress conditions since oxidative stress can promote stop codon read-through and the resulting translation into the 3΄UTR of key mRNAs may produce toxic and aberrant proteins.

When a stop codon is translocated into the ribosomal A-site, it is recognized by eRF1 (51). eRF1 activates hydrolysis of the ester bond between the completed polypeptide chain and the tRNA in the ribosomal P-site. eRF3 is a GTPase that associates with eRF1 and is essential for the termination reaction. Yeast Sup35 is well known for its ability to form prion aggregates known as [*PSI^+^*], which sequesters it away from its normal function in translation termination and elevated read-through of termination codons is therefore a well-known phenotype of the yeast [*PSI^+^*] prion (35). However, [*PSI*^+^] formation is an extremely rare event occurring at a frequency of ∼5 × 10^−4^ following exposure to H_2_O_2_ (36). Hence, the rare formation of [*PSI*^+^] in response to oxidative stress cannot account for aggregation and significant loss of Sup35 activity. Instead, we found that oxidative stress promotes amorphous aggregation of Sup35, which analogous to Sup35 amyloid formation, titrates Sup35 away from its normal function in translation termination. This was a relatively frequent event since ∼20% of Sup35 was sequestered into an insoluble aggregated form in response to H_2_O_2_ stress. A number of growth conditions can cause protein misfolding and amorphous aggregation including advanced age and environmental stresses such as oxidative stress (52,53). Highly abundant proteins such as Sup35 are prone to aggregation and it is thought that stress conditions such as oxidative stress causes aggregation by lowering the threshold for aggregate formation (54,55). Hence, alterations in the fidelity of translation termination due to amorphous aggregation of Sup35 may be an unanticipated consequence of aging and various stress conditions.

Our data indicate that NSD acts to protect cells against protein production when oxidative stress induced Sup35-aggregation depletes the pool of available soluble Sup35. We found that overexpressing Sup35 restored termination efficiency in agreement with the idea that aggregation of Sup35 titrates it away from its normal function in translation termination. We have tested one potential mechanism where the loss of Sup35 results in stop codon read-through and aberrant protein production. However, several previous studies have described additional functional roles for Sup35 which might be required during oxidative stress conditions. For example, there is evidence that release factors can discriminate against sense codons under normal conditions and Sup45–Sup35 may be capable of binding to stalled ribosomal complexes that contain a sense codon in the ribosomal A site (56,57). Sup35 is also involved in other cellular processes including mRNA decay through de-adenylation, chromosome segregation and cytoskeleton organization (58–60). Taken together, our data indicate that loss of Sup35 and the resulting increase in stop codon read-through, or loss of other Sup35-mediated processes, is detrimental to cells during oxidative stress conditions.

The evolutionarily conserved SKI complex functions in many cytoplasmic exosome-mediated pathways including 3΄–5΄-mRNA degradation, NSD and NMD (20,61–64). Loss of Hbs1 or Dom34 in a *ski7* mutant increased oxidant sensitivity suggesting that an overlapping role for Ski7 and the Dom34–Hbs1 complex in recognizing NSD substrates is required for oxidant tolerance. However, *hbs1 ski7* and *dom34 ski7* mutants were not as sensitive as a *ski2* mutant to H_2_O_2_ stress suggesting that oxidant sensitivity might not solely arise in a SKI complex mutant due to the inability to recognize and degrade NSD substrates. While PTCs are unlikely to be introduced into mRNAs as a result of oxidative stress, many mRNAs contain naturally occurring PTCs. Conditions which promote termination codon read-through such as oxidative stress may therefore cause the production of altered and potentially toxic proteins from such mRNAs. For example, whole genome sequencing of the 74D-694 yeast strain used in our study has shown that 22 genes encode potential premature stop codons which might affect the production of proteins involved in a variety of cellular processes (65). Interestingly, the 74D-694 strain background contains more PTCs compared with the BY4741 background and we found that *ski* mutants were more sensitive to oxidative stress in the 74D-694 background compared with the BY4741 background (compare Figure 3A and B). This is consistent with the presence of PTCs correlating with increased oxidant sensitivity, but it should be noted that other genomic differences between these different strain backgrounds may also influence oxidant sensitivity. It is also known that mRNA turnover is regulated in response to oxidative stress conditions and hence disrupting this process may also account for some oxidant sensitivity (10,11). Upf1, a key regulator of NMD, has also been shown to be required for the transcriptional induction of many oxidative stress-regulated genes in the fission yeast *Schizosaccharomyces pombe* and *upf1Δ* strains are sensitive to H_2_O_2_ stress (66). Further work will be required to understand the hypersensitivity of SKI complex mutants to oxidative stress. This will be important since RNA metabolism has many disease links and mutations in human exosome subunit genes have been linked with childhood-onset neurological diseases (67).
